# Deep-sea biodiversity at the extremes of the Salas y Gómez and Nazca ridges with implications for conservation

**DOI:** 10.1371/journal.pone.0253213

**Published:** 2021-06-30

**Authors:** Alan M. Friedlander, Whitney Goodell, Jonatha Giddens, Erin E. Easton, Daniel Wagner

**Affiliations:** 1 Pristine Seas, National Geographic Society, Washington, DC, United States of America; 2 Hawaiʿi Institute of Marine Biology, University of Hawaiʿi, Kāneʻohe, Hawaiʿi, United States of America; 3 Exploration Technology Lab, National Geographic Society, Washington, DC, United States of America; 4 Ecology and Sustainable Management of Oceanic Islands, Universidad Católica del Norte, Coquimbo, Chile; 5 School of Earth, Environmental, and Marine Sciences, University of Texas Rio Grande Valley, Brownsville, Texas, United States of America; 6 Conservation International, Center for Oceans, Arlington, VA, United States of America; Florida Institute of Technology, UNITED STATES

## Abstract

The Salas y Gómez and Nazca ridges are underwater mountain chains that stretch across 2,900 km in the southeastern Pacific and are recognized for their high biodiversity value and unique ecological characteristics. Explorations of deep-water ecosystems have been limited in this region, and elsewhere globally. To characterize community composition of mesophotic and deep-sea demersal fauna at seamounts in the region, we conducted expeditions to Rapa Nui (RN) and Salas y Gómez (SyG) islands in 2011 and Desventuradas Islands in 2013. Remote autonomous baited-cameras were used to conduct stationary video surveys between 150–1,850 m at RN/SyG (N = 20) and 75–2,363 m at Desventuradas (N = 27). Individual organisms were identified to the lowest possible taxonomic level and relative abundance was quantified with the maximum number of individuals per frame. Deployments were attributed with associated environmental variables (temperature, salinity, dissolved oxygen, nitrate, silicate, phosphate, chlorophyll-a, seamount age, and bathymetric position index [BPI]). We identified 55 unique invertebrate taxa and 66 unique fish taxa. Faunal community structure was highly dissimilar between and within subregions both for invertebrate (p < 0.001) and fish taxa (p = 0.022). For fishes, dogfish sharks (Squalidae) accounted for the greatest dissimilarity between subregions (18.27%), with mean abundances of 2.26 ± 2.49 at Desventuradas, an order of magnitude greater than at RN/SyG (0.21 ± 0.54). Depth, seamount age, broad-scale BPI, and nitrate explained most of the variation in both invertebrate (R^2^ = 0.475) and fish (R^2^ = 0.419) assemblages. Slightly more than half the deployments at Desventuradas (N = 14) recorded vulnerable marine ecosystem taxa such as corals and sponges. Our study supports mounting evidence that the Salas y Gómez and Nazca ridges are areas of high biodiversity and high conservation value. While Chile and Peru have recently established or proposed marine protected areas in this region, the majority of these ridges lie outside of national jurisdictions and are under threat from overfishing, plastic pollution, climate change, and potential deep-sea mining. Given its intrinsic value, this region should be comprehensively protected using the best available conservation measures to ensure that the Salas y Gómez and Nazca ridges remain a globally unique biodiversity hotspot.

## Introduction

As the largest habitat on Earth, the deep sea is remarkably underexplored and under-characterized [1, 2]. Consequently, many fundamental questions in deep-sea ecology remain unresolved [3, 4]. Although knowledge of deep-sea community composition and ecosystem functioning has advanced rapidly in recent decades, both in terms of the scale of sampling [5, 6] and large-scale data syntheses [5, 7–10], fundamental ecological data for much of the deep sea is lacking [11]. For example, a recent three-year, deep-sea exploration campaign in the Pacific found that fewer than 20% of the taxa could be identified, highlighting our knowledge limits of the deep ocean [12].

Lack of fundamental ecological knowledge, including what lives in the deep sea and how biodiversity and community composition are distributed, hinders efforts to map current species distributions and predict future distributions in a changing climate [13, 14], information which is highly relevant to planning management and conservation efforts. For example, habitat heterogeneity is thought to drive global patterns of species diversity, but relationships between environmental drivers and biological responses are still uncertain [4]. Further, maintaining well-connected populations, communities, and ecosystems is critical to design effective networks of marine protected areas (MPAs) [15, 16]. Lacking baseline knowledge and accurate modeling parameters hinders our ability to manage these ecosystems effectively [1, 3] and to meet the global conservation and sustainable development targets that define the next Decade of Ocean Science for Sustainable Development (2021–2030) [17]. Therefore, major questions to be addressed by the research community in support of conservation and sustainable development goals are to understand the diversity of life in the deep ocean, its distribution, and how populations and habitats are connected [3].

Bathyal and abyssal species diversity are among the highest of any ecosystem worldwide [18, 19]. Supporting this remarkably high biodiversity, the deep seafloor and water column is composed of a complex mosaic of physical, geological, and geochemical features [20, 21], including submarine mountain ranges. Though remote, these ecosystems are not isolated from anthropogenic impacts, which increasingly threaten the health of the deep ocean [20, 22–24].

Empirical studies have shown that seamounts and submarine ridges frequently harbor reef-building corals [25–27], and these habitats are well known for their high food availability, high proportion of endemic species [28, 29], and exceptional diversity of marine life [30–33]. These heterogeneous features support ecosystem connectivity by facilitating the dispersion of larvae between distant geographic areas and by serving as stepping stones and navigational marks for the movement of benthic and pelagic species and providing important feeding, resting, and spawning grounds [31–33]. The high variability of community composition within and among seamounts is related to environmental variation in these heterogeneous habitats [34, 35], providing evidence that each seamount is unique. Seamounts are vulnerable to anthropogenic disturbance (e.g., trawling) [31, 36] and often do not recover from intensive fishing pressure [37–39]; therefore, they are a high priority for conservation [27].

**Fig 1:**
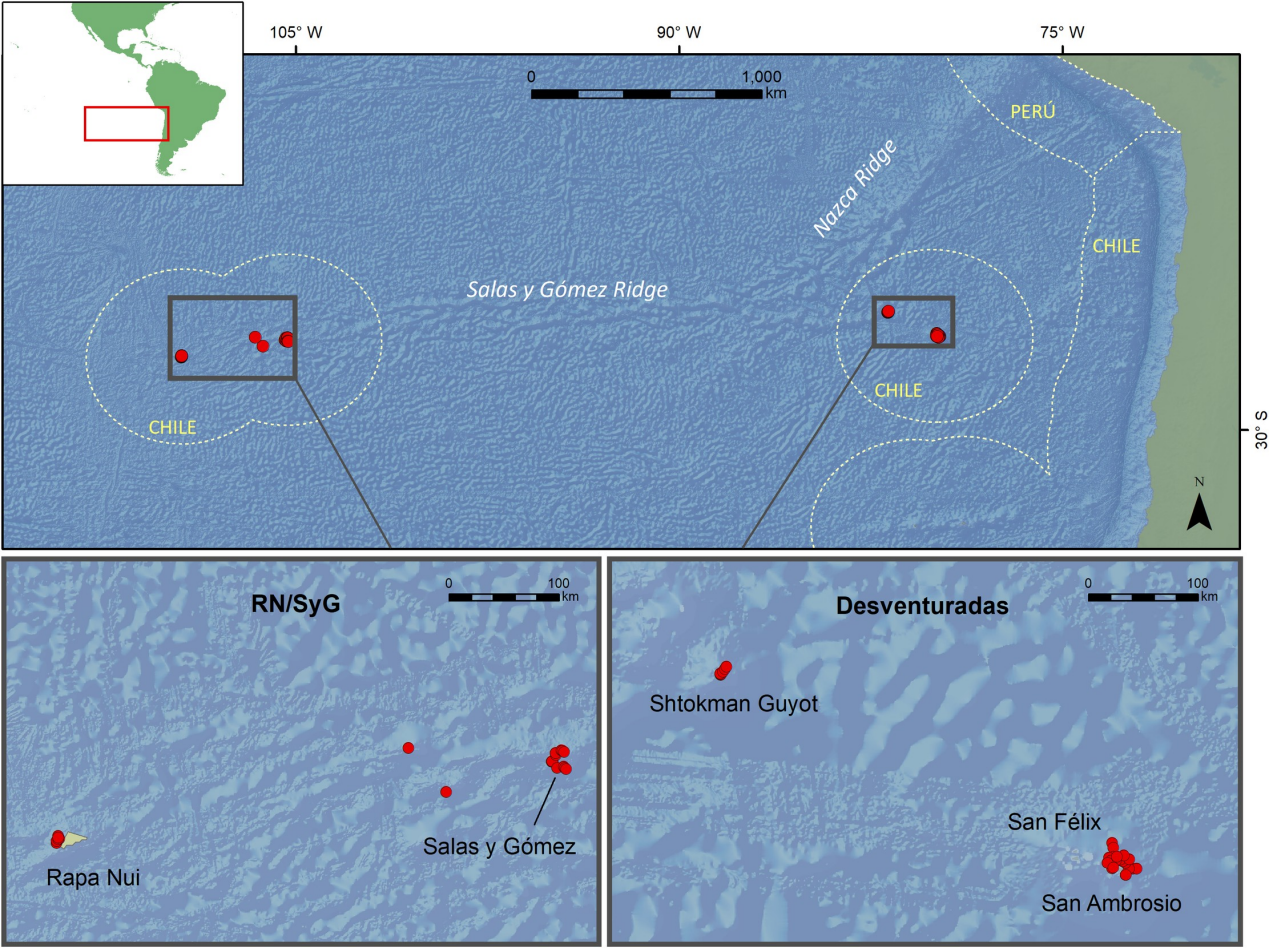
Deep-sea camera system deployments on the Salas y Gómez and Nazca ridges. Dotted lines depict exclusive economic zones (EEZs). Red dots depict camera deployment sites. Bathymetric data source: GEBCO Compilation Group (2020) GEBCO 2020 Grid (doi:10.5285/a29c5465-b138-234de053-6c86abc040b9).

Due to the dense aggregations of seamounts that comprise the Salas y Gómez and Nazca ridges in the southeastern Pacific, this region represents an important reservoir for global marine biodiversity [27, 29, 40, 41] (Fig 1). Despite its significance, there have been limited explorations of this region, especially in the deep sea, a pattern that is also evident for isolated islands globally [42–44] and in Pacific deep waters [45, 46]. The few deep-sea explorations along the Salas y Gómez Ridge have documented many species new to science (e.g., [47–49]). One campaign surveyed different seamounts across the ridge and found that the fauna on every seamount has a unique community composition, with nearly complete species turnover between opposite ends of the ridge [50]. Because of the high rate of species discoveries and uniqueness within and among seamounts, the marine fauna of this region likely contains numerous undiscovered species, providing an enormous opportunity for marine biodiversity research and high conservation value [51–53].

An expedition to Rapa Nui (i.e., Easter Island) and Salas y Gómez Island was conducted by the National Geographic Society’s Pristine Seas in February–March 2011 to provide a baseline assessment of the marine ecosystem at 75–2,363 m (Fig 1, [54]). In February and March 2013, National Geographic Society’s Pristine Seas also led an expedition to the Desventuradas Islands (Fig 1). Both expeditions resulted in the first systematic survey of these subregions, including its deep waters, using National Geographic Society Exploration Technology Lab’s deep-sea camera system [55]. These surveys provided a rare opportunity to visually examine the deep waters of the region at subregions that bookend the eastern and western portion of the Salas y Gómez Ridge, as well as the western portion of the Nazca Ridge (Fig 1).

The objectives of this research were to characterize biodiversity and community composition of deep-sea demersal fauna at seamounts surrounding Rapa Nui, Salas y Gómez Island, and the Desventuradas Islands to better understand deep-ocean biogeography and its environmental drivers in the region. We tested the hypotheses that biodiversity and faunal assemblage composition vary with location and environmental variables (e.g., depth, ocean chemistry, habitat complexity, geology). Our study represents the first characterization of deep-sea biodiversity spanning the east and west ends of the Salas y Gómez seamount chain, as well as the west end of the Nazca Ridge. By surveying remote deep-sea locations in a region of high conservation value, we provide important insights into patterns in biodiversity, connectivity, and its drivers for deep-sea ecosystem science and conservation, thereby advancing the global knowledge base for effective conservation and sustainable use.

## Methods

### Ethics statement

Data were collected by all authors in a collaborative effort. Non-invasive research was conducted, which included photographs and videos as described in the methods below. The Republic of Chile granted all necessary permissions to conduct this research. No vertebrate sampling was conducted and therefore no approval was required by any Animal Care and Use Committee. Our data are publicly available at Data Dryad: doi.org/10.5061/dryad.c866t1g6j.

### Study region

The Salas y Gómez and Nazca ridges are two adjacent volcanic seamount chains in the southeastern Pacific that stretch across more than 2,900 km of seafloor (Fig 1). The Nazca Ridge is adjacent to South America and stretches across approximately 1,100 km of seafloor between the eastern edge of the Salas y Gómez Ridge and the subduction zone off the Peruvian coast (Fig 1). A small northeastern section of the Nazca Ridge is in Peruvian national waters, whereas the majority of this ridge lies in areas beyond national jurisdiction (ABNJ). Stretching across roughly 1,600 km between the Nazca Ridge and Rapa Nui, the central portion of the Salas y Gómez Ridge lies within ABNJ. Both the western end around the islands of Rapa Nui and Salas y Gómez and the eastern end around San Félix and San Ambrosio (Desventuradas Islands) fall within Chile’s exclusive economic zone (EEZ) (Fig 1).

The Easter Island Ecoregion consists of Rapa Nui, Salas y Gómez, and the nearby seamounts [56]. Salas y Gómez Island (SyG) is considered part of Polynesia, making it the easternmost landmass of Polynesia and the most southeastern coral reef in the Pacific. Rapa Nui (RN) is considered one of the most isolated inhabited islands globally [57]. The island (total area of 166 km^2^) is located ~ 2,000 km east of Pitcairn Island, and ~3,700 km west of continental Chile. SyG is a small, emergent rock 0.15 km^2^ in area and 770 m in total length, with the highest point 30 m above sea level [58]. It is located ~390 km east of RN, and 3,210 km west of the Chilean mainland. These two islands are a part of the Salas y Gómez Ridge, and are the only places where the submarine mountain range rises above sea level [59, 60]. The several dozen seamounts in the Easter Island Ecoregion extend 2,232 km eastward where the Salas y Gómez Ridge joins the Nazca Ridge.

The Desventuradas Archipelago is located ~ 200 km southeast of the junction of the Salas y Gómez and Nazca ridges, rising from a depth of 4,000 m at just over 850 km from the Chilean coast. This archipelago consists of the islands of San Ambrosio, with a surface area of 2.2 km^2^, San Félix with a surface area of 1.4 km^2^, and the González and Roca Catedral islets (Saint Peterborough) (Fig 1). In total, the Chilean EEZ around the Desventuradas covers an area of 449,805 km^2^.

#### Data collection

Remote autonomous benthic landers with baited camera systems [55, 61] were used to conduct stationary video surveys at RN/SyG and the Desventuradas (Fig 1). These systems include high-definition cameras (Sony Handycam HDR-XR520V 12 megapixel) encased in a borosilicate glass sphere that are rated to 11,000 m depth. The camera is fixed at a 45° declination from the horizontal plane and has a 35 mm equivalent focal length of 29.8 mm, which results in a horizontal 62.27° and a vertical 37.53° angular field-of-view. Lighting at depth was achieved through a high-intensity LED array directed using external reflectors, and lighting intensity was constant among all deployments. Depth gauging was conducted using an external pressure sensor. The anchor line extended 2 m from the seafloor to optimize the field of view to both capture large mobile scavenging megafauna and allow for identification of organisms.

The deep-sea camera system is positively buoyant, resulting in an ascent rate of 0.5 ms^−1^, and weighted with a 22 kg external weight, resulting in a descent rate of 1.5 ms^−1^. The weight is released by burn wire, activated using onboard battery voltage, and the camera system was located for recovery by communication of an onboard VHF transmitter and locating antennae, with backup location via communication with the ARGOS satellite system.

Up to three deep-sea camera systems were simultaneously deployed between 22 February– 4 March, 2011 at 20 stations between 150–1,850 m depth ($\bar{X}$ = 1,104.6 ± 433.6) along the island slopes of SyG and RN and the slopes of two seamounts west of SyG (Fig 1). At Desventuradas, 27 successful deployments were conducted between 10–26 February, 2013 at 75–2,363 m depth ($\bar{X}$ = 755.8 ± 649.2) (Fig 1). Cameras were deployed with a minimum distance of 500 m from another deployment location, and each camera was baited with chopped frozen fish and deployed for 1 to 5 hours with an average footage per deployment of 112 min at Desventuradas and 129 min at RN/SyG. Deployments targeted geological features and specific depths based on available bathymetry. Sampling design aimed to represent a gradient of depths away from a feature (e.g., island, seamount), and thus deployments generally occurred in a line radiating out from the shallow depths of a feature to deeper waters.

The substrata for each camera deployment were classified as hard or soft, based on video footage. Seafloor type was defined as soft if the seafloor observed during the deployment consisted of ≥80% sediment (sands and muds), and hard if <80% of the seafloor observed during the deployment was composed of sediment. Hard habitats included rock, cobbles, pebbles, gravel, biogenic material (including live coral), coral rubble, and shell material.

#### Biological data processing

Annotations were made from the video footage based on standardized taxonomic nomenclature according to the World Register of Marine Species (WoRMS). Video files were stored in Tator, which is a cloud-based collaborative video annotation platform developed by CVisionAI (http://cvisionai.com/). Frame grabs of representatives of each species were taken in Tator for archival and identification purposes.

**Fig 2:**
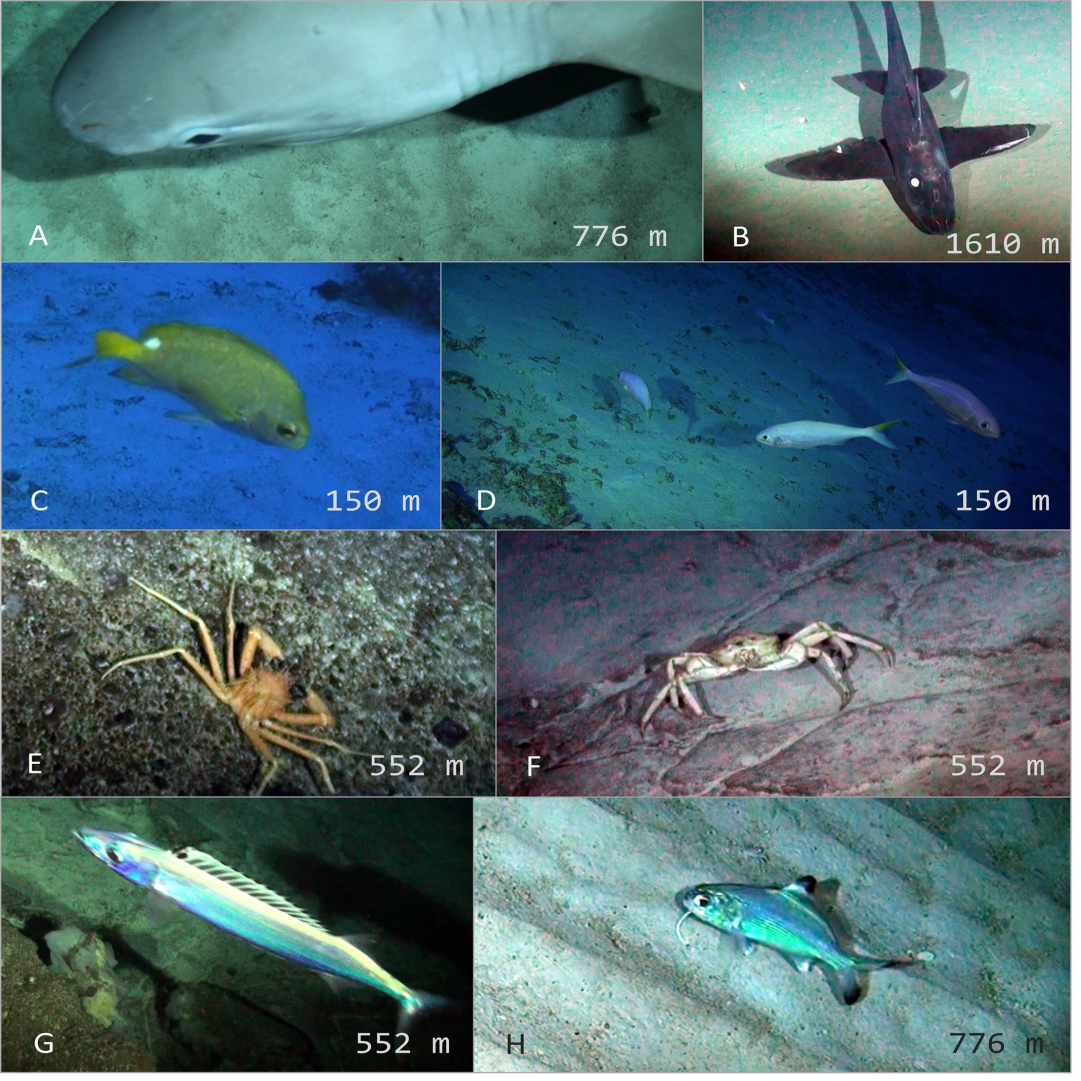
Representative and unique taxa observed with deep-sea camera systems around Rapa Nui and Salas y Gómez islands. (A) *Hexanchus griseus* (B) *Hydrolagus melanophasma* (C) Chromis mamatapara* (D) *Parapristipomoides squamimaxillaris* (E) Homolidae, *Yaldwynopsis* sp. (F) Geryonidae (G) *Rexea* sp. (H) Polymixia salagomeziensis*. *endemic species.

**Fig 3:**
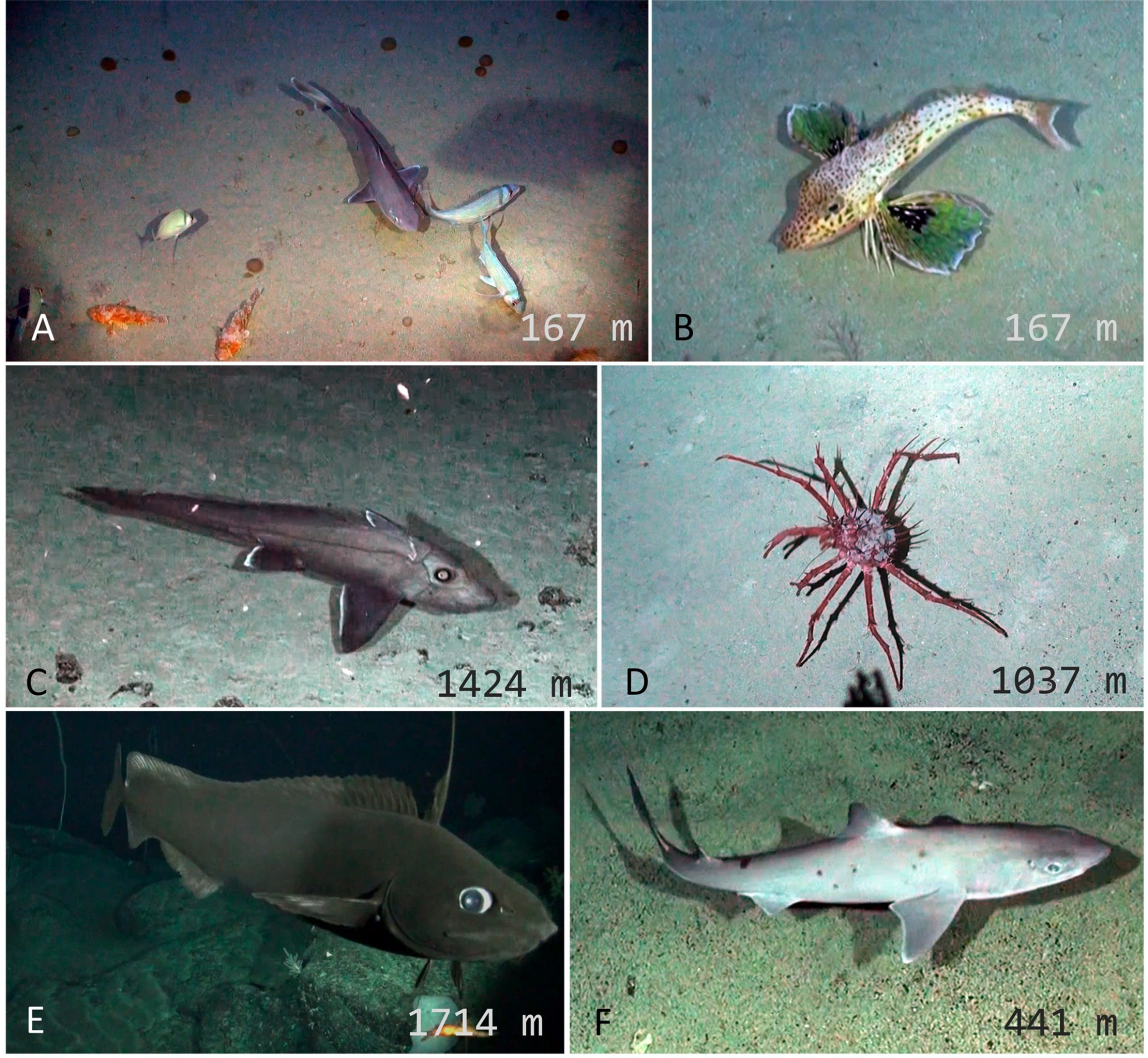
Representative and unique taxa observed with deep-sea camera systems at Desventuradas Islands. (A) Nemadactylus gayi*, Scorpaena orgila*, *Squalus* sp. (B) *Pterygotrigla picta* (C) *Hydrolagus* sp. (D) Lithodidae (E) *Antimora rostrata* (F) *Squalus* sp. *endemic species.

Individuals were identified to the lowest possible taxonomic level using the Hawaii Underwater Research Laboratory (HURL) database ([62]; http://www.soest.hawaii.edu/HURL/HURLarchive/guide.php), the NOAA Office of Ocean Exploration Benthic Deepwater Animal identification Guide (https://oceanexplorer.noaa.gov/okeanos/animal_guide/animal_guide.html), FishBase [63], regional guides and reports of trawl data from the Salas y Gómez and Nazca ridges [64–67], and input from regional taxonomic experts (Figs 2 and 3). The maximum number of individuals of each species in a single video frame (MaxN), rather than a total tally per deployment, was recorded to ensure that individuals were not double counted [68]. Taxa were classified as vulnerable marine ecosystem (VME) taxa based on recent studies [69, 70].

#### Geospatial data processing

To characterize samples by the physicochemical factors of a deployment location, each deployment was attributed with its associated ecological marine unit (EMU) [71]. EMUs are ecological divisions of the 3D marine environment, representing physically and chemically distinct water volumes, from sea surface to seafloor. The divisions are based on statistical clustering of six marine environmental parameters (temperature, salinity, dissolved oxygen, nitrate, phosphate, and silicate) (S1 Table). The environmental attribute data used in the statistical clustering are based on a set of objectively analyzed climatological fields of *in situ* long-term historical average data from the 2013 World Ocean Atlas version 2 (https://www.nodc.noaa.gov/OC5/woa13/) and represent the means of the decadal averages from a 57-year climatology of the six parameters. The database is a point-mesh grid that includes over 52 million points that depict the global ocean in x, y, and z dimensions. The data points were statistically clustered based on the six environmental parameters using a k-means clustering algorithm to identify environmentally distinct regions in the water column. The resulting 37 distinct EMUs partition the ocean environment into ecologically relevant regions and are a valuable tool in exploring drivers of biological distributions.

This resulting EMU dataset has a spatial resolution of ¼° × ¼° (~27 km × 27 km at the equator) in the horizontal dimension and was accessed from ESRI EMU online (http://esri.maps.arcgis.com/home/group.html?id=6c78a5125d3244f38d1bc732ef0ee743). EMU numbers, and values for oceanographic variables derived from the World Ocean Atlas 2013 database (temperature, salinity, dissolved oxygen, %O_2_ saturation, nitrate, silicate, phosphate, chlorophyll-a) were extracted from the EMU dataset using the Extract Multi Values to Points tool in the ArcGIS Spatial Analyst toolbox. EMUs and associated environmental variables vary across horizontal space as well as with depth, represented by the EMU dataset as a 3D point grid. Deployments were attributed with the respective EMU at the given sample depth, thus capturing depth-specific variations in physicochemical factors. Values for particulate organic carbon (POC) flux to the seafloor were obtained from Lutz et al. [72] via Watling et al. [73].

Seamount data were obtained from the Global Seamount Database [74]. Distance to the nearest seamount and the number of seamounts near sample sites were derived for a 30 km radius around deployment locations using the Buffer tool in ArcGIS Pro. Age of the underlying crust [75] was extracted for the seamount nearest each deployment location, using the Extract Multi Values to Points tool, as an indication of seamount age. Bathymetric position index (BPI) is an index of bathymetric heterogeneity that highlights areas of topographic anomalies and is used to identify benthic features (e.g., ridges and valleys) [76]. It is a continuous metric of the elevation of a pixel relative to other pixels in a landscape [77]. BPI values at or near zero represent consistent bathyscapes, either flat areas or areas of constant slope, whereas sharp peaks or pits are represented by higher absolute values [77, 78]. BPI was calculated in ArcMap 10.2 using the Benthic Terrain Modeler extension [79]. Input bathymetric data used was Global Multi-Resolution Topography (GMRT) [80] from the Marine Geoscience Data System (www.marine-geo.org), with spatial resolution of 3 arcseconds (~90 m). The GMRT dataset, available through OpenTopography (https://opentopography.org/), is a compilation of edited multibeam sonar datasets collected worldwide. The data for our study region includes swaths of mapping data throughout the region, intersecting and surrounding deployment locations.

Broad-scale BPI was calculated using an inner radius of 25 cells and an outer radius of 50 cells. Fine-scale BPI was derived with inner and outer radii of 1 and 10 cells, respectively. Both broad- and fine-scale BPI have been shown to be associated with variations in marine faunal communities [76, 81], including those of the deep ocean [45, 82].

### Statistical methods

Due to the numerical dominance of Amphipoda in our samples, they were excluded from all analyses, except for the comparisons of number of taxa and analyzed separately. Total MaxN per deployment was calculated as the sum of MaxN for all taxa for that deployment. Species diversity was calculated from the Shannon-Weaver Diversity Index [83]:$H\prime =-\sum (p_{i}lnp_{i})$, where p_i_ is the proportion of all individuals counted that were of species i. Pielou’s evenness was calculated as: J = H´/ln(S), where S is the total number of species present.

A principal components analysis was used to examine environmental variables between subregions. Environmental variables in this analysis included depth, temperature, salinity, dissolved oxygen, %O_2_ saturation, nitrate, silicate, phosphate, chlorophyll-a, broad-scale BPI, fine-scale BPI, POC, crust age, nearest seamount, and number of seamounts within 30 km of the sample sites. Data were Box-Cox transformed, centered, and standardized prior to analysis. The absolute values of fine-scale and broad-scale BPI metrics were used since peaks and troughs both represent measures of habitat complexity. A comparison of environmental variables between subregions and habitats was conducted using permutation-based multivariate analysis of variance (PERMANOVA) with a Euclidean distance matrix.

Deployment depth was examined as a predictor variable in explaining the variability in invertebrate taxa richness, fish taxa richness, and fish MaxN between subregions and habitats (i.e., hard and soft) using generalized linear models (GLMs) with a Poisson distribution with a log-link function. The GLMs for fish diversity and evenness used a Gamma distribution with a log-link function. Comparison of Amphipoda abundance between subregions was tested using a Wilcoxon rank sum test. Comparisons of invertebrate and fish assemblage structure between subregions and habitats was also performed using PERMANOVA with Bray-Curtis similarity matrices. Similarity percentages analysis (SIMPER) was used to examine differences in invertebrate and fish assemblages between subregions using Bray-Curtis similarity analysis of hierarchical agglomerative group average clustering.

Relationships between environmental variables and invertebrate and fish assemblages were analyzed using distance-based linear models (DistLM) and redundancy analysis (dbRDA). Linear models are appropriate for these data because a preliminary detrended correspondence analysis showed short gradient lengths (<2 SD) [84]. The dbRDA introduces a series of explanatory (environmental) variables and resembles the model of multivariate multiple regression, allowing us to determine what linear combinations of these environmental variables determine the gradients. These analyses were conducted using Bray-Curtis similarity matrices on MaxN Class or Order-level invertebrate data and fish family-level data. MaxN invertebrate data were ln(x+1) transformed and MaxN fish data were 4^th^-root transformed prior to analyses to conform to the assumptions of normality and homogeneity of variances [85]. Normality was tested using a Shapiro-Wilk W test (p < 0.05) while a Bartlett’s test (p < 0.05) was used to examine homogeneity of variance. We determined the model with environmental drivers that best explained variation in invertebrate and fish assemblages using a stepwise routine that employed 9999 permutations based on Akaike’s Information Criterion with second order correction (AICc) selection criterion. Environmental variables that had high correlations (r > 0.95) were removed prior to analysis. Those removed were temperature, silicate, %O_2_ saturation, and phosphate. All statistical analyses were conducted using JMP Pro 15 and Primer 6.0.

## Results

### Environmental variables

Of the 20 camera deployments conducted around RN/SyG, 40% (N = 8) were located on hard bottom habitat. In contrast, only 15% (n = 4) of the 27 deployments at Desventuradas were located on this habitat type. Deployment depth was not significantly different between subregions, habitats, or their interaction (all GLMs p > 0.05).

**Fig 4:**
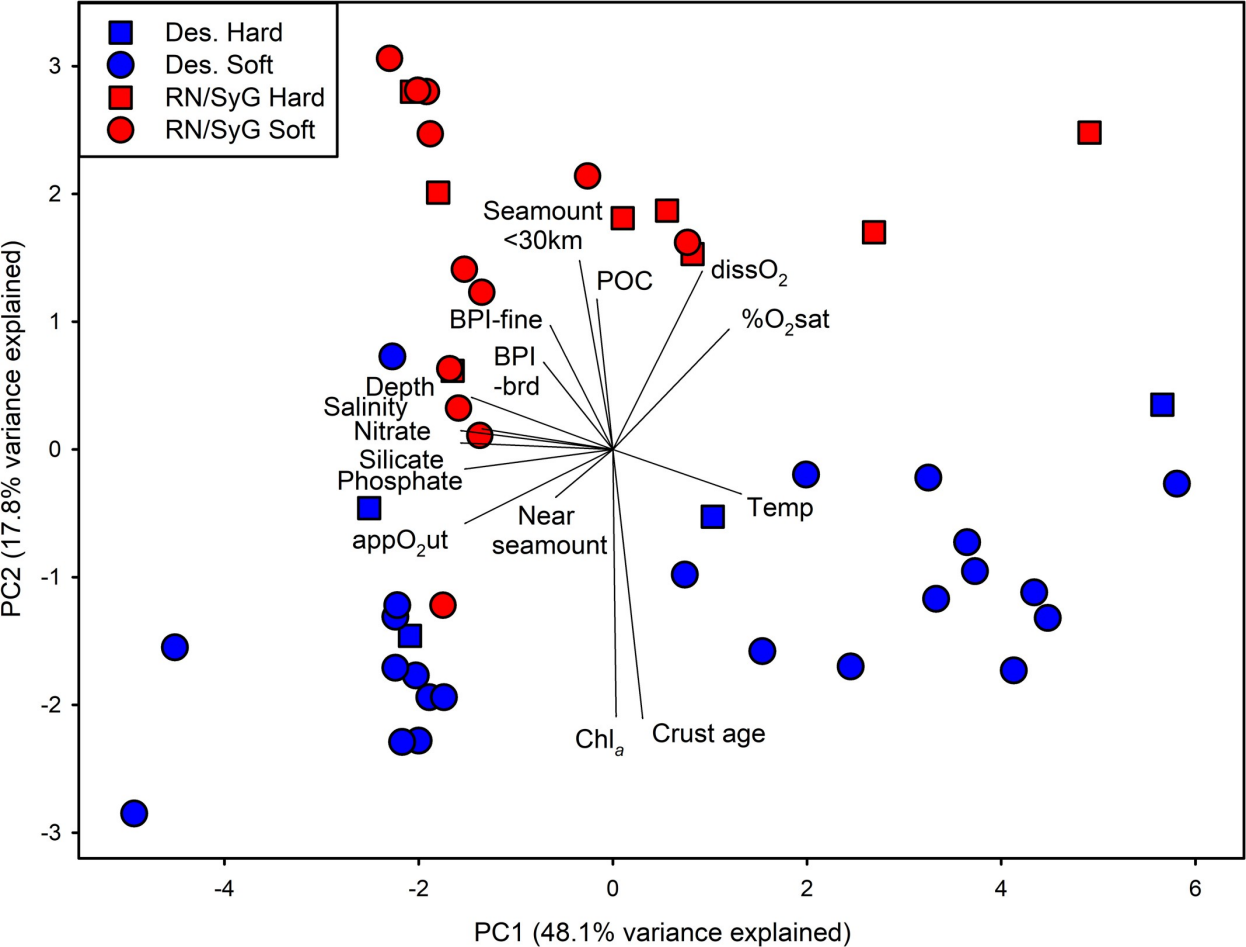
Principal Components Analysis (PCA) of environmental variables at camera deployment sites. Data were Box-Cox transformed prior to analysis. Des. = Desventuradas.

Subregions were well separated in ordination space based on environmental variables (Fig 4). Environmental variables were significantly different between subregions (PERMANOVA pseudo-F_1,43_ = 5.456, p < 0.001) but not between habitats (PERMANOVA pseudo-F_1,43_ = 1.524, p = 0.188) or their interaction (PERMANOVA pseudo-F_1,43_ = 0.699, p = 0.552). PC1 accounted for 48.1% of the variation in environmental variables, with the major loadings being silicate (-0.349), nitrate (-0.342), phosphate (-0.340), apparent oxygen utilization (-0.340), and depth (-0.335). PC2 accounted for an additional 17.8% of the variation in environmental variables, with the major loadings being crust age (-0.504), chlorophyll-a (-0.503), number of seamounts within 30 km pf the sample sites (0.358), and dissolved O_2_ (0.329). Nitrate, phosphate, silicate, salinity, and depth were all highly correlated with one another and inversely related to temperature. Subregions separated out along PC2 with sites at Desventuradas being older geologically with higher chlorophyll-a. Deployments at RN/SyG had more seamounts within 30 km of the sample sites and higher measures of habitat complexity (BPI) at both fine and broad scales.

### Invertebrate assemblages

There were 56 invertebrate taxa from 5 phyla, 12 classes, 13 orders, and 22 infra-orders or families identified on camera deployments (S2 Table). There were no significant differences in the number of invertebrate taxa between subregions, habitats, or their interactions (all GLMs p > 0.05). Amphipoda were the most common and abundant taxa observed. They were present on 95% of the deployments at RN/SyG ($\bar{X}$ = 68.50 ± 108.67) and 48% of the deployments at Desventuradas ($\bar{X}$ = 11.89 ± 23.9). In addition, Amphipoda were 5.8 times more abundant at RN/SyG compared to Desventuradas (Wilcoxon = 2.273, p = 0.023).

Invertebrate assemblage structure was significantly different between subregions (PERMANOVA pseudo-F_1,43_ = 12.848, p < 0.001) but not between habitats (PERMANOVA pseudo-F_1,43_ = 1.927, p = 0.096) or their interaction (PERMANOVA pseudo-F_1,43_ = 0.186, p = 0.951). The invertebrate assemblages were more similar among deployments at RN/SyG (45.98%) compared to Desventuradas (21.37%), with average dissimilarity between subregions equal to 85.52%. Decapoda accounted for the greatest dissimilarity between subregions (60.83%), with abundances at RN/SyG ($\bar{X}$ = 22.40 ± 25.91) more than 15 times higher compared to Desventuradas ($\bar{X}$ = 1.48 ± 1.74). The dominant components of this order were shrimp from the families Benthesicymidae, Aristeidae, and Sergestidae. Anthozoa accounted for an additional 15.1% of the dissimilarity between subregions, with abundances at Desventuradas ($\bar{X}$ = 5.26 ± 11.96) more than 20 times higher than at RN/SyG ($\bar{X}$ = 0.25 ± 0.72).

### Vulnerable marine ecosystems

**Fig 5:**
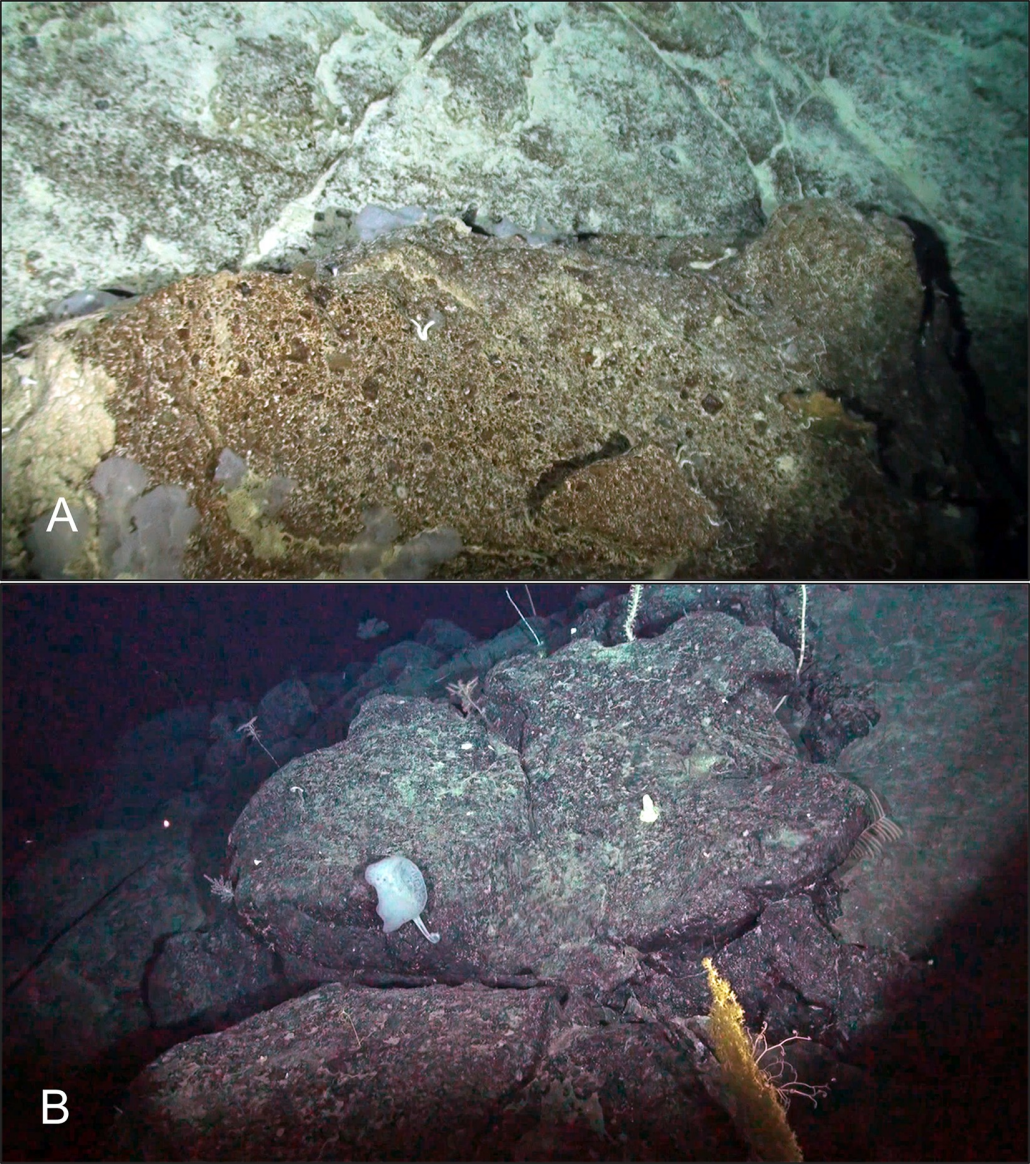
Vulnerable marine ecosystem indicator taxa, including deep-sea corals and sponges, observed on deep-sea camera deployments along the Salas y Gómez and Nazca ridges. (A) Sala y Gómez—552 m (B) Desventuradas– 1,714 m.

Consistent with other studies, colonial Anthozoa and Porifera were classified as vulnerable marine ecosystem (VME) taxa ([79] and references therein). Within Anthozoa, there were several VME taxa, including Pennatulacea, Antipatharia, Actiniaria, and Alcyonacea (Fig 5). Within Porifera, Hexactinellida and Demospongiae were classified as VME taxa. More than half the deployments at Desventuradas (n = 17, 63%) had VME taxa present, while only 3 (15%) of the deployments at RN/SyG had VME taxa present.

### Invertebrate-habitat relationships

**Fig 6:**
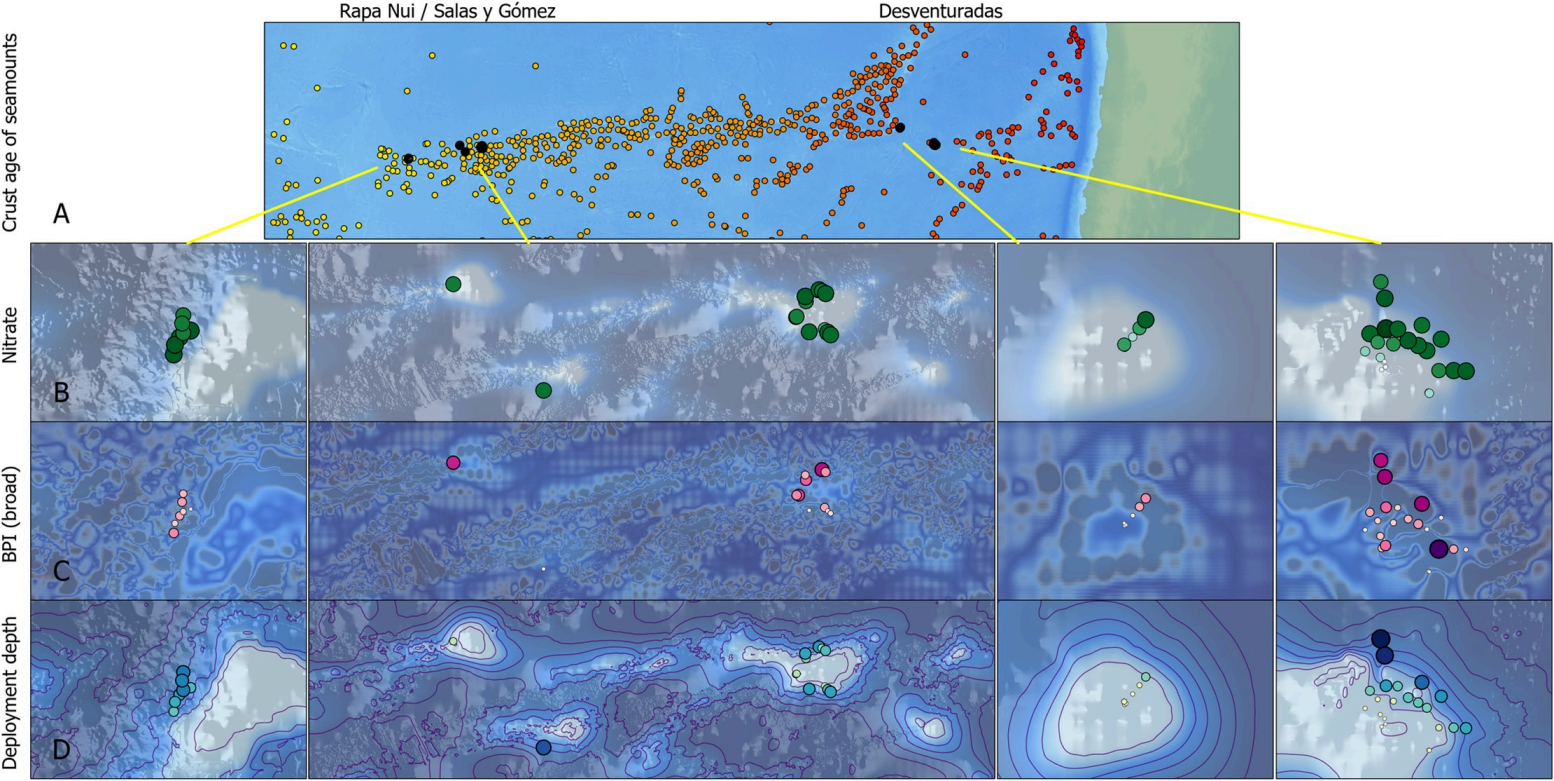
Environmental variables at the deep-sea camera system deployment locations. (A) Crust age (younger → older: yellow → red) was extracted for the nearest seamount to each deployment location. (B) Nitrate levels; bubbles depict nitrate levels at deployment location and depth (low → high nitrate: small/light → big/dark). (C) Absolute broad-scale bathymetric position index (BPI); bubbles depict absolute BPI values at deployment location (low → high BPI: small/light → big/dark), basemap depicts absolute BPI values of region (low →high: dark blue → gray). (D) Depth of each deployment; bubbles depict depth at deployment location (small/light → big/dark: shallow → deep), basemap depicts bathymetry (deep → shallow: dark blue → white). Purple lines in Panel D are 500 m contour lines. Bathymetric data source: Global Multi-Resolution Topography (GMRT) [80, www.marine-geo.org].

**Fig 7:**
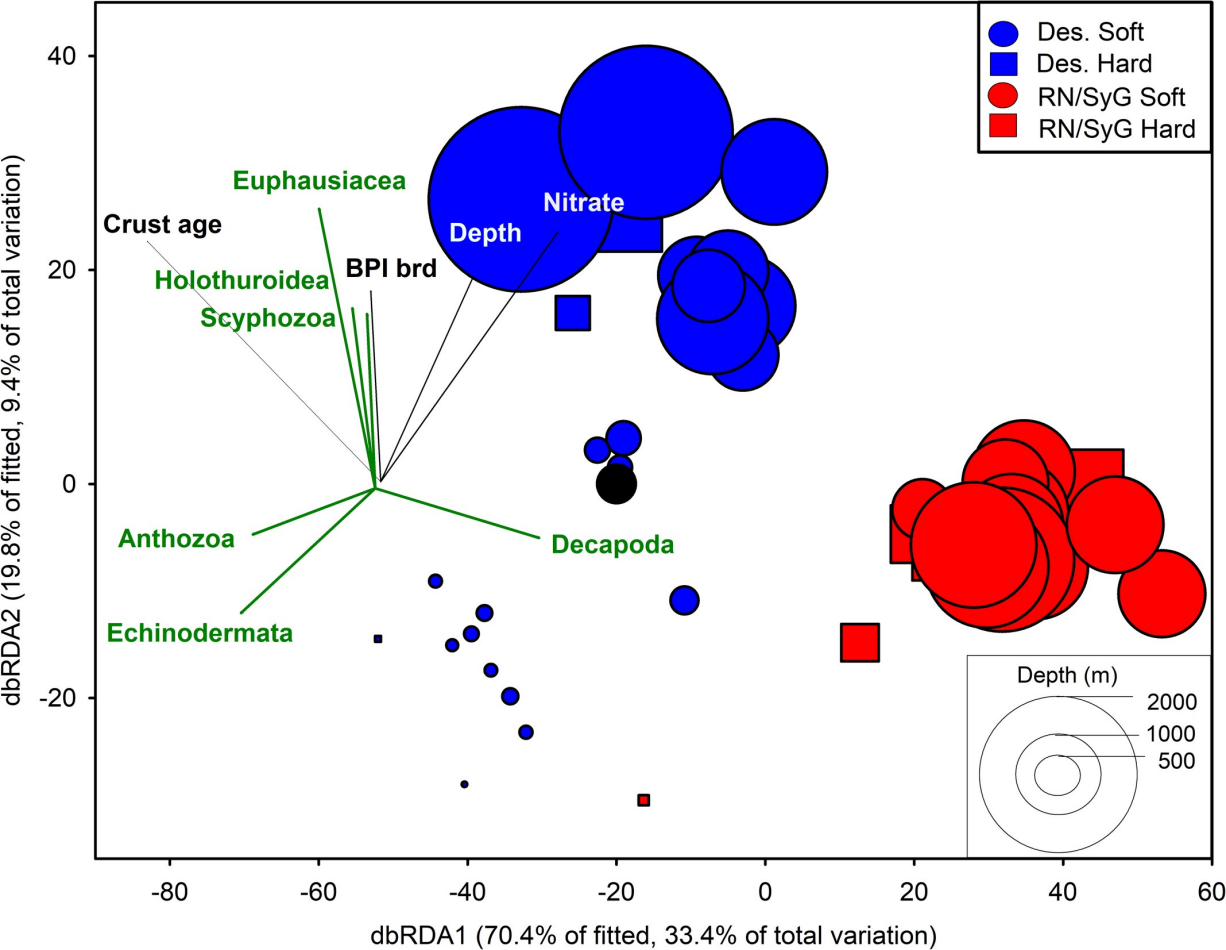
Distance-based redundancy analysis (dbRDA) ordination to investigate the relationship between the environmental variables and invertebrate assemblages. Bray-Curtis similarity matrix based on ln(x+1) transformed MaxN higher level invertebrate taxa. Symbol sizes are proportional to depth.

The best distance-based linear model solution between environmental variables and invertebrate assemblage structure included crust age, nitrate, broad-scale BPI, and depth (AIC_c_ = 349.17, R^2^ = 0.475) (Table 1, Fig 6). Crust age accounted for 26.75% of the variation followed by nitrate (13.96%), broad-scale BPI (3.84%), and depth (2.95%). Deployments by subregions were well-separated in ordination space, and sites within Desventuradas had a greater spread and lower concordance compared to RN/SyG (Fig 7). dbRDA1 accounted for 70.35% of the fitted variation and 33.41% of the total variation, with crust age (*ρ* = -0.779) and nitrate (*ρ* = 0.559) being the major drivers along this axis (Table 1). dbRDA2 accounted for 19.75% of the fitted variation and 9.38% of the total variation, with all four variables contributing to this axis. Decapoda (*ρ* = 0.518), Echinodermata (*ρ* = *-*0.448), and Anthozoa (*ρ* = *-*0.407) were most highly correlated with dbRDA1. Euphausiacea (*ρ* = 0.612), Holothuroidea (*ρ* = 0.391), and Scyphozoa (*ρ* = 0.385) were most closely correlated with dbRDA2.

**Table 1:** Distance-based linear model for invertebrate assemblages and environmental variables.

Sequential tests							Multiple partial correlations	
Variable	AICc	SS(trace)	Pseudo-F	P	Prop.	Cumul.	dbRDA1	dbRDA2
Crust age	357.63	31603	16.432	0.001	0.267	0.267	-0.779	0.546
Nitrate	349.98	16493	10.359	0.001	0.140	0.407	0.559	0.558
BPI broad	349.23	4531	2.974	0.019	0.038	0.445	-0.036	0.425
Depth	349.17	3487	2.361	0.052	0.030	0.475	0.282	0.457

Multiple partial correlations are the relationships between dbRDA coordinate axes and orthonormal X variables. Prop. = proportion of variation explained. Cumul. = cumulative proportion variation explained.

### Fish assemblages

**Fig 8:**
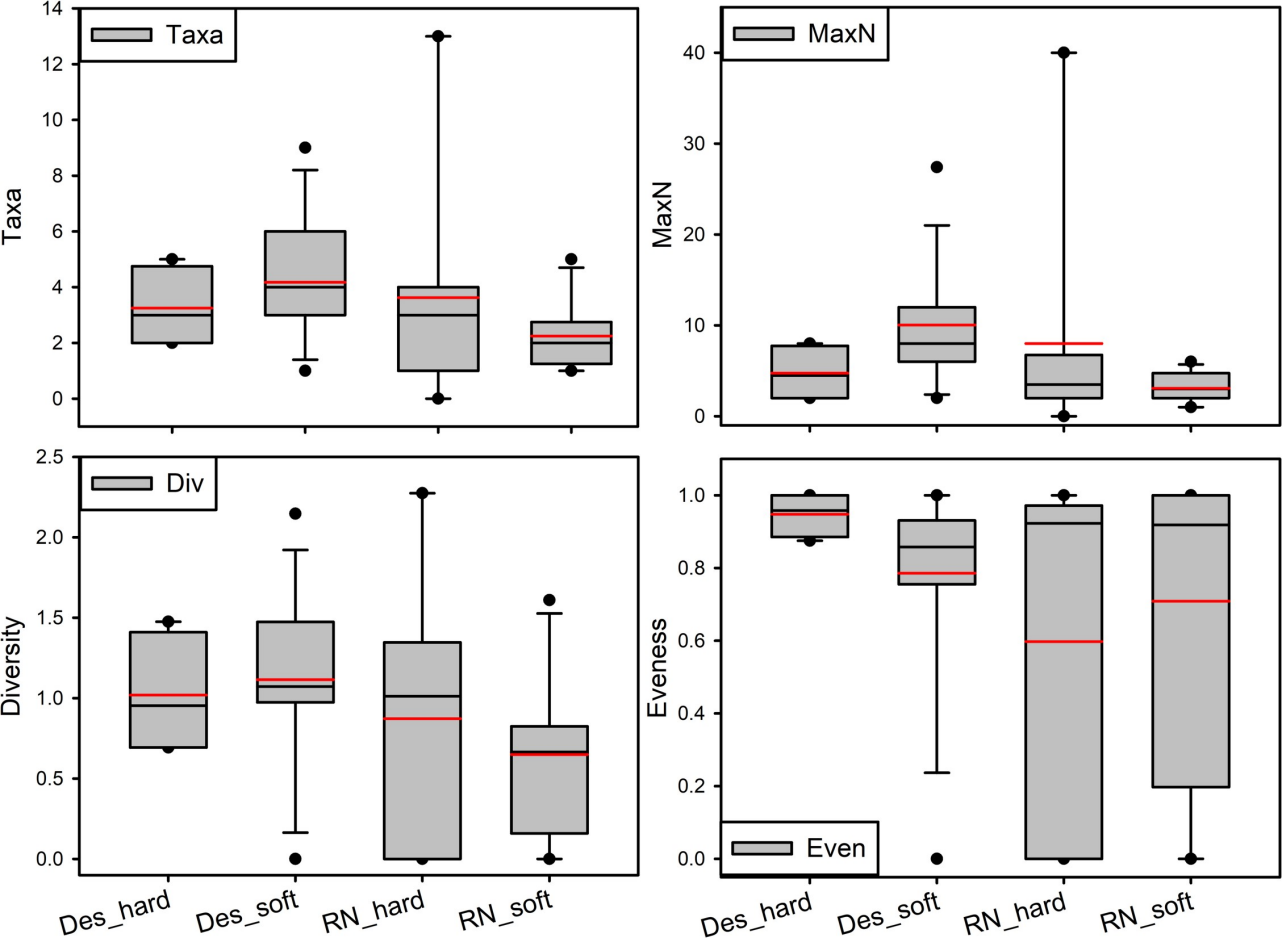
Fish assemblage characteristics (taxa richness, MaxN, diversity, and evenness) at Desventuradas and RN/SyG on hard and soft-bottom habitats. Box plots showing median (black line), mean (red line), upper and lower quartiles, and 5th and 95th percentiles.

There were 61 fish taxa from 17 orders and 39 families identified on camera deployments (S3 Table). Most families only had one or two representative taxa except for Etmopteridae, Macrouridae, and Serranidae, which each had three taxa per family. There were no significant differences in the taxonomic richness, diversity, or evenness between subregions, habitats, or their interactions (Fig 8, all GLMs p > 0.05). MaxN was significantly different between subregions (GLM *X*^2^_1,43_ = 4.156, p = 0.042) and for the interaction between region and habitat (GLM *X*^2^_1,45_ = 34.027, p < 0.001) but not between habitats alone (GLM *X*^2^_1,43_ = 0.414, p = 0.520). MaxN was highest in Desventuradas soft bottom habitats and lowest in RN/SyG soft bottom habitats.

Fish assemblage structure was significantly different between subregions (PERMANOVA pseudo-F_1,43_ = 2.482, p = 0.022) but not between habitats (PERMANOVA pseudo-F_1,43_ = 1.431, p = 0.184) or their interaction (PERMANOVA pseudo-F_1,43_ = 1.974, p = 0.059). The average dissimilarity between subregions was 91.01%. Squalidae accounted for the greatest dissimilarity between subregions (18.27%), with abundances at Desventuradas ($\bar{X}$ = 2.26 ± 2.49) an order of magnitude greater than at RN/SyG ($\bar{X}$ = 0.21 ± 0.54) (Table 2). Moridae accounted for an additional 7.98% of the dissimilarity between subregions, with abundances at Desventuradas ($\bar{X}$ = 0.63 ± 1.04) 42% higher than at RN/SyG ($\bar{X}$ = 0.26 ± 0.56). Abundances of Macrouridae at RN/SyG ($\bar{X}$ = 0.53 ± 1.02) were 43% times higher than at Desventuradas ($\bar{X}$ = 0.37 ± 0.69) and accounted for an additional 7.31% of the dissimilarity between subregions. Deployments within subregions were highly dissimilar, with the average similarity among deployments at RN/SyG equal to 14.47% and the average similarity among deployments at Desventuradas equal to 15.99%.

**Table 2:** SIMPER for fish families most responsible for the percent dissimilarities between subregions using Bray-Curtis similarity analysis of hierarchical agglomerative group average clustering.

Families	Des	RN/SyG	Diss.	Contrib%	Cum%
Squalidae	2.26 (2.49)	0.21 (0.54)	16.63 (0.89)	18.27	18.27
Moridae	0.63 (1.04)	0.26 (0.56)	7.27 (0.69)	7.98	26.25
Macrouridae	0.37 (0.69)	0.53 (1.02)	6.65 (0.68)	7.31	33.56
Halosauridae	0.41 (0.97)	0.37 (0.68)	6.25 (0.63)	6.87	40.43
Synaphobranchidae	0.30 (0.67)	0.47 (0.70)	6.18 (0.64)	6.80	47.22
Pentacerotidae	1.04 (2.9)	-	5.65 (0.39)	6.21	53.43
Gempylidae	-	0.58 (0.90)	5.57 (0.55)	6.12	59.55
Cheilodactylidae	0.85 (3.05)	-	3.12 (0.27)	3.42	62.97
Carangidae	0.28 (1.53)	0.16 (0.97)	2.97 (0.32)	3.27	66.24
Etmopteridae	0.22 (0.42)	-	2.50 (0.47)	2.75	68.99
Chimaeridae	0.07 (0.27)	0.16 (0.50)	2.45 (0.40)	2.69	71.68
Sebastidae	0.37 (1.18)	-	2.33 (0.30)	2.56	74.24
Ophidiidae	0.30 (1.20)	-	2.19 (0.26)	2.41	76.65
Serranidae	0.30 (1.35)	0.53 (2.29)	2.14 (0.35)	2.35	79.00
Labridae	0.15 (0.77)	0.16 (0.69)	1.58 (0.23)	1.74	80.73
Lutjanidae	-	0.58 (2.29)	1.53 (0.31)	1.68	82.41
Triglidae	0.26 (0.53)	-	1.45 (0.45)	1.59	84.00
Polymixiidae	0 (0)	0.16 (0.50)	1.39 (0.29)	1.52	85.53
Monocentridae	0.22 (0.64)	-	1.21 (0.34)	1.32	86.85
Myctophidae	0.07 (0.27)	0.05 (0.23)	1.12 (0.35)	1.23	88.08
Alepocephalidae	0.07 (0.27)	-	1.05 (0.24)	1.15	89.23
Hexanchidae	-	0.11 (0.46)	0.98 (0.22)	1.08	90.31

Average dissimilarity = 91.08%. Values are average MaxN with one standard deviation of the mean in parentheses. DES = Desventuradas, Diss. = dissimilarity, Contrib% = percent contribution to dissimilarity, Cum% = cumulative percent contribution to dissimilarity.

### Fish-habitat relationships

**Fig 9:**
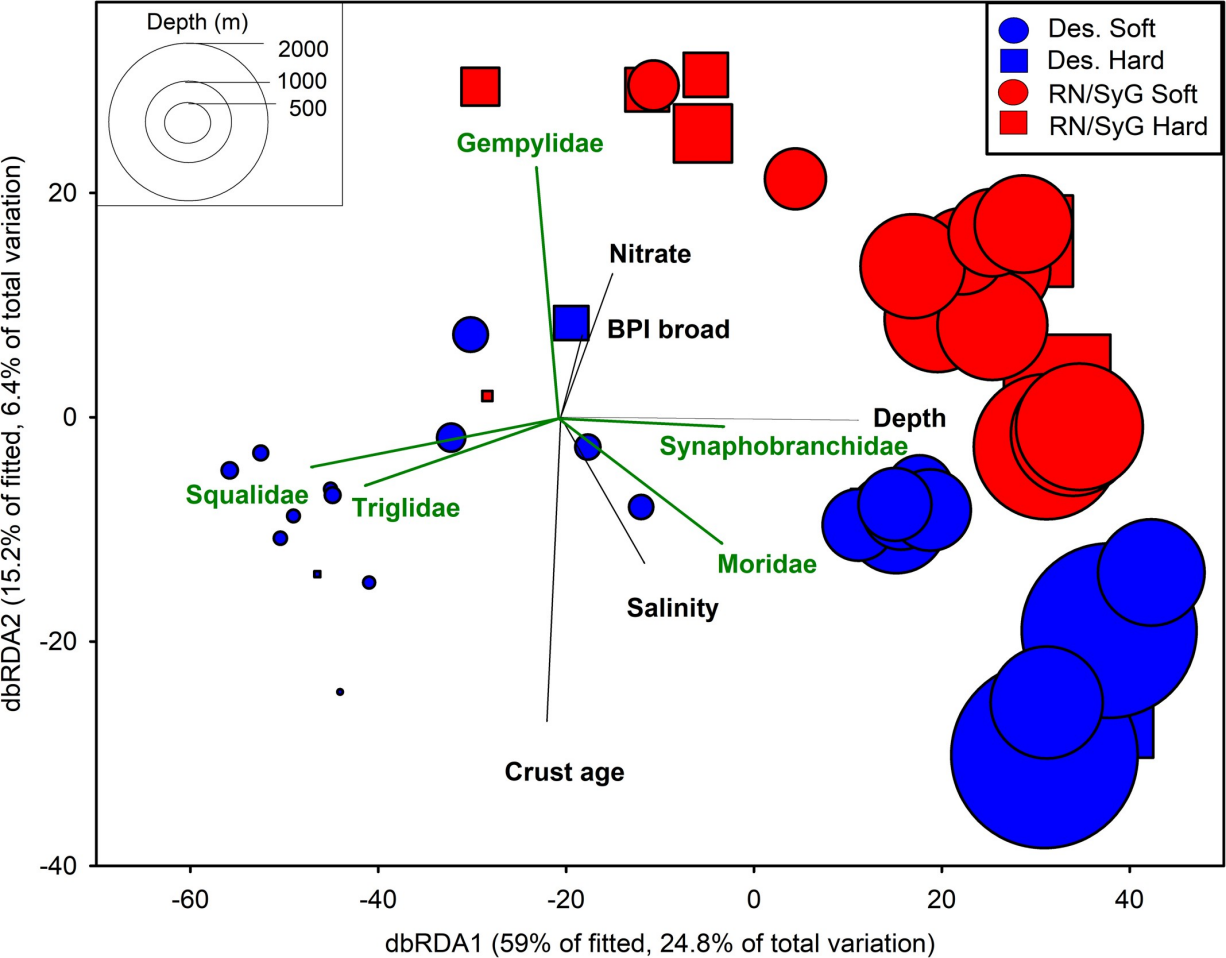
Distance-based redundancy analysis (dbRDA) ordination to investigate the relationship between the environmental variables and fish assemblages. Bray-Curtis similarity matrix based on 4^th^ root-transformed MaxN higher level fish taxa. Symbol sizes are proportional to depth.

The best distance-based linear model solution between environmental variables and fish assemblage structure included depth, salinity, crust age, nitrate, and broad-scale BPI (AIC_c_ = 367.83, R^2^ = 0.419) (Table 3). Depth accounted for 22.68% of the variation followed by salinity (5.79%), crust age (4.83%), nitrate (4.58%), and broad-scale BPI (4.12%). Deployments by subregions were well-separated in ordination space and sites within Desventuradas had a greater spread and lower concordance compared to RN/SyG (Fig 9). dbRDA1 accounted for 59.04% of the fitted variation and 24.79% of the total variation. Depth (*ρ* = 0.947) was the major driver of dbRDA1. dbRDA2 accounted for 15.16% of the fitted variation and 6.37% of the total variation, with the major drivers being crust age (*ρ* = -0.810), followed by nitrate (*ρ* = -0.385) and salinity (*ρ* = -0.384).

**Table 3:** Distance-based linear model for fish assemblages and environmental variables.

Sequential tests							Multiple partial correlations	
Variable	AICc	SS(trace)	Pseudo-F	P	Prop.	Cumul.	dbRDA1	dbRDA2
Depth	371.18	39264	12.903	0.001	0.227	0.227	0.947	-0.006
Salinity	369.90	10021	3.479	0.001	0.058	0.285	0.264	-0.384
Crust age	369.08	8370	3.044	0.002	0.048	0.333	-0.033	-0.81
Nitrate	368.34	7924	3.020	0.003	0.046	0.379	0.166	0.385
BPI broad	367.83	7136	2.842	0.007	0.041	0.420	0.076	0.217

Multiple partial correlations are the relationships between dbRDA coordinate axes and orthonormal X variables. Prop. = proportion of variation explained. Cumul. = cumulative proportion variation explained.

Squalidae (ρ = -0.797), Triglidae (ρ = -0.627), Moridae (ρ = 0.519), and Synaphobranchidae (ρ = 0.509) were most highly correlated with dbRDA1. Gempylidae (ρ = 0.678), Sternoptychidae (ρ = 0.407), Polymixiidae (ρ = 0.350) and Moridae (ρ = 0.330) were most closely correlated with dbRDA2. The snake mackerel family (Gempylidae), primarily *Rexea* spp., was correlated with hard bottom sites at RN/SyG. Spiny dogfish sharks (Squalidae—*Squalus* spp.) and sea robins (Triglidae—*Pterygotrigla picta*) were most closely correlated with shallow sites at Desventuradas, while morid cod (Moridae–primarily *Antimora rostrata*) were correlated with deeper sites at Desventuradas. Cutthroat eels (Synaphobranchidae–primarily cf. *Synaphobranchus affinis*) were correlated with deeper sites at both RN/SyG and Desventuradas.

## Discussion

### Community assemblage and habitat relationships

Faunal community structure based on our camera deployments was highly dissimilar between and within subregions. Our findings are consistent with previous studies that note that every seamount along the ridges may have a unique faunal composition [50, 65, 66]. Seamount systems such as the Salas y Gómez and Nazca ridges, which are geographically and hydrographically isolated due to the influence of the region’s current systems, are likely to have developed highly endemic taxa and ecosystems [66, 86]. The Salas y Gómez and Nazca ridges comprise over 110 seamounts, which represent roughly 41% of seamounts found in the southeastern Pacific [41]. Given the high rate of discovery of new species, this region may well harbor many more undiscovered marine species [51, 52].

Depth, crust age, broad-scale BPI, and nitrate explained the most variation in both invertebrate and fish assemblages on our camera deployments, and our findings are largely consistent with other studies [45, 82, 87]. Community composition on seamounts is depth stratified, reflecting environmental gradients that correlate with depth, such as temperature, oxygen concentration, food availability, and pressure [32]. The high habitat heterogeneity on seamounts, quantified by BPI, combined with the biological effects of depth, increases local biodiversity by providing neighboring areas with different ecological niches, which helps explain the high dissimilarity of invertebrate assemblages within and between seamounts of a region [88]. In our study, crust age was highly correlated with region, as the seamounts become progressively older moving eastward along the ridges from 2 million years on the western portion of the chain to over 27 million years towards the northeastern end [60, 89].

### An endemism and biodiversity hotspot

The nearshore marine ecosystems around the emergent islands of RN/SyG and Desventuradas are known to have some of the highest marine endemism anywhere on Earth [29, 54, 67]. Recent genetic studies have confirmed that Rapa Nui is an endemism hotspot: a location for the emergence of small-range endemic fishes (likely resulting from founder-event speciation), a route of dispersion for larger-range endemics, and a stepping stone for the diversification of some groups [90].

Our study supports evidence that the biodiversity hotspot and role of ecosystem connectivity extends beyond the shallow coastal waters, and into the deep sea surrounding RN/SyG and Desventuradas. For example, *Chromis mamatapara* is a recently described damselfish from around Rapa Nui and is believed to be endemic to the mesophotic ecosystems of Rapa Nui, Salas y Gómez, and nearby seamounts. We recorded two individuals of *Chromis mamatapara* at Salas y Gómez Island in 150 m (Fig 2). Several species are known to occur at both RN/SyG and Desventuradas islands, which highlights the connectivity between these emergent islands along the ridge. The moray eel *Gymnothorax bathyphilus*, which was previously known only from Rapa Nui and Desventuradas, was observed at Salas y Gómez Island in 150 m. Similarly, Gay’s morwong (*Nemadactylus gayi*) is known only from the Desventuradas and Juan Fernández islands and was observed at three sites around Desventuradas with up to 14 individuals in a single frame. The beardfish (*Polymixia salagómeziensis*) is endemic to the Salas y Gómez Ridge and we recorded individuals at two locations near Salas y Gómez Island between 640 and 644 m. *Plectranthias nazcae* is endemic to the Nazca Ridge and was recorded at Desventuradas in 167 m. We observed several invertebrate species that are endemic to the region, including two crabs (*Chaceon chilensis* and *Paromola rathbuni*), one lobster (*Jasus frontalis*), and one sea urchin (*Stereocidaris nascaensis*). Collectively, these findings highlight that the region is a global hotspot of endemic of species, and that the ridges are important for species connectivity.

### Threats

#### Fishing

A number of commercially exploited fisheries species were observed during our study. These included two endemic decapods: the Juan Fernández lobster (*Jasus frontalis*), which was only observed at one station at Desventuradas in 156 m, and the Chilean golden crab (*Chaceon chilensis*), which was observed on 44% of the deployments around Desventuradas between 201 and 1,352 m. These species are currently harvested at sustainable levels by the small-scale artisanal fishers from the nearby Juan Fernández Archipelago [91, 92]. The recently created Nazca-Desventuradas Marine Park and Mar de Juan Fernández Marine Park were strongly promoted by the Juan Fernández community to preserve their traditional lobster fishing and to exclude industrial fishing [93]. In addition, the Juan Fernández trevally (*Pseudocaranx chilensis*) and Gay’s morwong (*Nemadactylus gayi*) are both endemic to the Desventuradas and Juan Fernández archipelagos, where they are important bait for the lobster fishery [94]. However, these endemic species are not protected on the seamounts in adjacent ABNJ and are susceptible to overexploitation by industrial-scale fisheries.

Immediate threats to the region are from large-scale foreign fishing fleets, primarily from China, who accounted for over 72% of the total fishing effort in this region between 2012 and 2020 [53]. Seamount habitats worldwide are often heavily impacted by fishing efforts as deep-sea fish species aggregate and often spawn on seamounts and can be slow to recover from disturbance. For example, heavy fishing efforts in the 1960s to the 1980s on seamounts on the far northwestern Hawaiian Ridge and Emperor Seamount Chain resulted in the largest quantity of fish and invertebrate biomass removed from any documented seamount fishery in the world [38]. These fisheries collapsed by the early 1980s, and after 40 years of protection, the habitats are only showing signs of limited recovery [39]. Further, seamounts are often dominated by deep-sea corals and sponges that are generally slow growing and extremely long lived. For example, a black coral species taken at 450 m off O‘ahu, Hawai‘i is the oldest skeletal-accreting marine organism known (4,265 yrs) and the oldest colonial organism yet found on Earth [95].

Over 73% of the Salas y Gómez and Nazca ridges are in ABNJ without protections from fishing. Globally, ABNJ account for only 4.2% of annual marine capture fisheries production, with most species destined for upscale markets in developed, food-secure countries, such as Japan, the European Union, and the United States, suggesting high seas fisheries play a negligible role in ensuring global food security [96]. Deep-sea and high seas fishing often produces net economic benefits only due to subsidies as most of the world’s largest fishing fleets would be unprofitable without subsidies and low labor costs [97]. Closing the high seas would be catch-neutral, while reducing inequality in the distribution of fisheries benefits among the world’s maritime countries by 50% [98].

### Climate change

It is predicted that in the next 20–40 years, the seafloor of this region will experience increases in temperature, acidification, decreases in dissolved oxygen, and declining export of particulate organic carbon [24, 99]. Portions of this region are already extremely nutrient [100] and oxygen-poor [51], so the global threat of climate change is especially heightened in this region and in need of management intervention to protect ecosystem structure and function. The numerous endemic species in this region are at high risk of extinction from climate change, as they have limited ranges, small thermal safety margins, and unique habitat and environmental requirements [29, 101, 102]. In addition, primary productivity and fisheries production are projected to decline throughout the region over the next several decades due to climate change [103], and these changes will impact the entire ecosystem and require a precautionary approach to management of human use.

### Conservation opportunities

Numerous previous studies have noted the exceptional natural and cultural significance of the Salas y Gómez and Nazca ridges (reviewed in [53]). In 2014, at the 12th Meeting of the Conference of the Parties [104], the Salas y Gómez and Nazca ridges were recognized as an ecologically or biologically significant marine area (EBSA). The eastern part of the Nazca Ridge is part of the most productive marine ecosystem in the world and is one of the region’s most unique underwater habitats [29, 105]. Seamounts on the Salas y Gómez and Nazca ridges host a high abundance and diversity of unique organisms and provide important habitat and migration corridors for protected megafauna such as blue whales, leatherback turtles, and sharks; important fisheries species such as swordfish (*Xiphias gladius*) and Chilean jack mackerel (*Trachurus murphyi*); and a diversity of other ecologically important species such as habitat-forming deep and shallow-water corals [27, 41, 106–110]. The results of this study provide additional evidence that the Salas y Gómez and Nazca ridges are of high natural significance and conservation value. Our surveys documented numerous VME and endemic taxa. In addition, some of the endemic taxa were documented on both ends of the Salas y Gómez Ridge, supporting previous conclusions that the ridges are important for connectivity as they provide migration corridors and ecological stepping stones for many species [104].

Chile has already protected the portions of the Salas y Gómez and Nazca ridges that fall within its jurisdictions through various MPAs, including the Rapa Nui Multiple-Use Coastal Marine Protected Area, the Motu Motiro Hiva Marine Park, and the Nazca-Desventuradas Marine Park. While these conservation efforts represent important conservation advances for the region, collectively they only cover 23.8% of the area recognized as an EBSA. Although the portion of the Nazca Ridge that lies within Peru’s national waters has been proposed for increased protections [111], it remains unprotected, highlighting an immediate opportunity for Peru to expand its ocean conservation efforts. To date, Peru has protected only a small fraction of its national waters (< 0.5%) and fallen short of achieving its Aichi Biodiversity Target 11 commitment of protecting 10% of its national waters by 2020.

While the recent efforts by Chile and Peru to establish or propose MPAs in the national waters of this region provide important advances, these efforts could be undermined if surrounding ecosystems in ABNJ cannot be properly conserved [53]. Over 73% of the ridges fall outside national jurisdictions, where they are unprotected and under threat from overfishing, climate change, plastic pollution, and potential seabed mining. This region has been recognized as one of the most important to protect on the high seas globally [27, 112–117]. While the surveys of our study all occurred within Chilean waters of the region, it is highly likely that high seas waters between our study subregions also provide critical habitats and ecological stepping stones for many of the species documented during our surveys. For example, seafloor topography data indicates that there are many seamounts in ABNJ of this region [21], which are well known to provide highly suitable habitats for many of the demersal fish, coral, and sponge taxa recorded in our surveys [69]. Furthermore, recently developed habitat suitability models, indicate that highly suitable habitat for VME taxa is widespread in ABNJ of the Salas y Gómez and Nazca ridges [118]. Most large-scale features (e.g., seamounts, guyots, ridges, and escarpments) in this region provide suitable habitats for deep-water corals and sponges and all our survey sites with VME taxa present (n = 20) were in areas of high habitat suitability based on these distribution models. Given the importance of VME taxa in deep-sea ecosystem functioning and their slow recovery rates from disturbance, these features should be proactively protected from bottom fishing and seabed mining.

### Caveats

The deep ocean presents many challenges in exploration and assessment given the extreme environmental conditions, remote locations, and paucity of existing data on which to base targeted studies [1]. For example, determining habitat type *a priori* was not possible, resulting in an unbalanced sampling design in our study. Expanded development and accessibility of seafloor mapping datasets would allow for more informed sampling design and enable more balanced representation of different habitat types [119].

Our study is based on opportunistic sampling to provide baseline observations at two remote and underexplored locations. Like all baited-camera methods, our deep-sea camera system is specialized to measure the relative abundance of mobile megafauna that are attracted to bait [61, 120, 121]. However, species’ absences may be less reliably measured by opportunistic point video surveys [122]. Further, our deep-sea camera system is designed to optimize the survey field of view and the ability to identify distinguishing characteristics of mobile taxa (e.g., pectoral and anal fins and head characteristics) [61]. However, without physical sampling, species-level identifications can be difficult or impossible to determine from video alone. For example, in the Desventuradas, a new species of Squalidae may have been present, but a physical specimen would be required for confirmation. In most cases in our study, taxonomic resolution was achieved to the family level for fishes and order level for invertebrates, which was sufficient for statistical comparisons of assemblage-level differences among locations.

Moreover, for baited camera methods, small sessile fauna (e.g., meiofanua, infauna) in the background of a frame can be difficult to confidently identify, therefore this method typically focuses on assessments of mobile megafauna [61, 120, 123]. However, our study recorded numerous VME taxa, therefore we included sessile fauna in our analyses of invertebrate assemblages as these taxa indicate areas that are of high conservation value.

## Conclusion

Our study supports mounting evidence that the Salas y Gómez and Nazca ridges have high biodiversity and unique ecological characteristics. The rich deep-water communities that inhabit this region provide a lifeline for marine biodiversity in the Pacific, and sustain productivity with surrounding waters, representing a sentinel site for ecosystem connectivity across large ocean corridors. However, the consequences of continued or increased stressors on this marine region are severe. While our study did not focus on characterizing stressors, previous studies have concluded that this region is threatened by plastic pollution, climate change, industrial fishing, and seabed mining [53]. With informed, effective, and strong conservation measures, we can ensure the Salas y Gómez and Nazca ridges remain a globally unique biodiversity hotspot.

## Supporting information

S1 Table Summary statistics for environmental variables between regions deep-sea camera deployments along the Salas y Gómez and Nazca ridges. (DOCX)

S2 Table Invertebrate taxa observed on deep-sea camera deployments along the Salas y Gómez and Nazca ridges. VME = vulnerable marine ecosystem taxa. Msp = morpho-species. Freq. Des = frequency of occurrence Desventuradas (n = 27). Freq. Rn/SyG = frequency of occurrence Rapa Nui/Salas y Gómez (n = 20).
(DOCX)

S3 Table Vertebrate taxa observed on deep-sea camera deployments along the Salas y Gómez and Nazca ridges. Msp = morpho-species. * = endemic species.
(DOCX)
