# Supplementary material for: Deep-sea biodiversity at the extremes of the Salas y Gómez and Nazca ridges with implications for conservation
Source: PLoS One. 2021 Jun 30;16(6):e0253213. doi: 10.1371/journal.pone.0253213 (PMC8244922; doi:10.1371/journal.pone.0253213)
Supplement: S1 Table — (DOCX) [file pone.0253213.s001.docx]

**S1 Table**. Summary statistics for environmental variables between regions deep-sea camera deployments along the Salas y Gómez and Nazca ridges.

| Expedition | RN/SyG | | | | Desventuradas | | | |
| --- | --- | --- | --- | --- | --- | --- | --- | --- |
|  | Mean (sd) | Min | Max | Mean (sd) | | Min | Max |  |
| Max depth | 1104.65 (433.61) | 150 | 1849 | 755.81 (649.22) | | 75 | 2363 |  |
| Temperature | 4.2 (3.41) | 2.07 | 17.98 | 6.92 (4.45) | | 1.66 | 15.06 |  |
| Salinity | 34.49 (0.1) | 34.31 | 34.64 | 34.46 (0.12) | | 34.31 | 34.7 |  |
| AppO2ut | 3.8 (0.96) | 0.46 | 4.47 | 3.68 (1.36) | | 0.39 | 5.64 |  |
| DissO2 | 3.54 (0.53) | 3.04 | 5.02 | 3.15 (0.76) | | 1.82 | 5.27 |  |
| Nitrate | 34.21 (7.94) | 2.43 | 39.71 | 28.59 (11.3) | | 5.07 | 42.26 |  |
| %O2sat | 48.59 (12.18) | 39.48 | 91.82 | 47.77 (16.55) | | 24.79 | 93.32 |  |
| Phosphate | 2.41 (0.52) | 0.32 | 2.68 | 2.2 (0.65) | | 0.74 | 3.03 |  |
| Silicate | 71.76 (32.94) | 1.53 | 115.48 | 56.82 (49.42) | | 2.78 | 162.24 |  |
| Chl*_a_* | 0.04 (0.01) | 0.03 | 0.09 | 0.24 (0.39) | | 0.07 | 1.99 |  |
| Bpigmrt fine | 0.45 (19.76) | -32 | 52 | -0.96 (11.35) | | -29 | 33 |  |
| Bpigmrt brd | 19.35 (112.34) | -158 | 217 | 24.74 (116.58) | | -233 | 302 |  |
| Bpigmrt fine ABS | 12.55 (15) | 0 | 52 | 6.59 (9.2) | | 0 | 33 |  |
| Bpigmrt brd ABS | 93.75 (61.34) | 10 | 217 | 84.81 (82.2) | | 0 | 302 |  |
| Seamounts within 30k | 4.4 (2.64) | 0 | 8 | 2.89 (1.01) | | 2 | 4 |  |
| Nearest seamount | 0.09 (0.07) | 0.02 | 0.35 | 0.08 (0.03) | | 0.04 | 0.17 |  |
| Crust age | 7.77 (2.3) | 4.4 | 9.4 | 34.6 (0.38) | | 33.9 | 34.8 |  |
