# Supplementary material for: Deep-sea biodiversity at the extremes of the Salas y Gómez and Nazca ridges with implications for conservation
Source: PLoS One. 2021 Jun 30;16(6):e0253213. doi: 10.1371/journal.pone.0253213 (PMC8244922; doi:10.1371/journal.pone.0253213)
Supplement: S2 Table — VME = vulnerable marine ecosystem taxa. Msp = morpho-species. Freq. Des = frequency of occurrence Desventuradas (n = 27). Freq. Rn/SyG = frequency of occurrence Rapa Nui/Salas y Gómez (n = 20). (DOCX) [file pone.0253213.s002.docx]

**S2 Table.** Invertebrate taxa observed on deep-sea camera deployments along the Salas y Gómez and Nazca ridges. VME = vulnerable marine ecosystem taxa. Msp = morpho-species.

| Phylum | Class | Order | Infraorder/Family | Taxa | VME |
| --- | --- | --- | --- | --- | --- |
| Arthropoda | Malacostraca | Amphipoda |  |  | N |
| Arthropoda | Malacostraca | Decapoda |  |  | N |
| Arthropoda | Malacostraca | Decapoda | Anomura/ Parapaguridae | *Sympagurus* sp. | N |
| Arthropoda | Malacostraca | Decapoda | Aristeidae |  | N |
| Arthropoda | Malacostraca | Decapoda | Benthesicymidae | *Benthesicymus* sp. | N |
| Arthropoda | Malacostraca | Decapoda | Brachyura |  | N |
| Arthropoda | Malacostraca | Decapoda | Brachyura/ Homolidae | *Maridae* cf *cyrtomaia* | N |
| Arthropoda | Malacostraca | Decapoda | Brachyura/ Homolidae | *Yaldwynopsis* sp. | N |
| Arthropoda | Malacostraca | Decapoda | Geryonidae | *Chaceon chilensis* | N |
| Arthropoda | Malacostraca | Decapoda | Homolidae | *Paromola rathbuni* | N |
| Arthropoda | Malacostraca | Decapoda | Lithodidae |  | N |
| Arthropoda | Malacostraca | Decapoda | Munididae |  | N |
| Arthropoda | Malacostraca | Decapoda | Nematocarcinidae | *Nematocarcinus* sp. | N |
| Arthropoda | Malacostraca | Decapoda | Paguroidea |  | N |
| Arthropoda | Malacostraca | Decapoda | Palinuridae | *Jasus frontalis* | N |
| Arthropoda | Malacostraca | Decapoda | Palinuridae | *Projasus bahamondei* | N |
| Arthropoda | Malacostraca | Decapoda | Pandalidae |  | N |
| Arthropoda | Malacostraca | Decapoda | Pandalidae | *Plesionika* sp. | N |
| Arthropoda | Malacostraca | Decapoda | Sergestidae |  | N |
| Arthropoda | Malacostraca | Euphausiacea |  |  | N |
| Arthropoda | Malacostraca | Euphausiacea | Euphausiidae |  | N |
| Arthropoda | Malacostraca | Stomatopoda |  |  | N |
| Arthropoda | Pycnogonida |  |  |  | N |
| Cnidaria | Anthozoa |  |  |  | Y |
| Cnidaria | Anthozoa |  |  | Cnidaria | Y |
| Cnidaria | Anthozoa | Actiniaria |  |  | Y |
| Cnidaria | Anthozoa | Alcyonacea |  |  | Y |
| Cnidaria | Anthozoa | Alcyonacea | Gorgoniidae |  | Y |
| Cnidaria | Anthozoa | Antipatharia |  |  | Y |

**S2 Table.** Continued. Invertebrate taxa observed on deep-sea camera deployments along the Salas y Gómez and Nazca ridges. VME = vulnerable marine ecosystem taxa. Msp = morpho-species.

| Phylum | Class | Order | Infraorder/Family | Taxa | VME |
| --- | --- | --- | --- | --- | --- |
| Cnidaria | Anthozoa | Antipatharia | Antipathidae | *Stichopathes* | Y |
| Cnidaria | Anthozoa | Antipatharia | Cladopathidae |  | Y |
| Cnidaria | Anthozoa | Antipatharia | Schizopathidae | *Bathypathes* sp. | Y |
| Cnidaria | Anthozoa | Pennatulacea |  |  | Y |
| Cnidaria | Anthozoa | Pennatulacea | Pennatulidae |  | Y |
| Cnidaria | Scyphozoa |  |  |  | N |
| Echinodermata |  |  |  |  | N |
| Echinodermata | Asteroidea |  |  |  | N |
| Echinodermata | Asteroidea | Paxillosida |  |  | N |
| Echinodermata | Crinoidea | Comatulida |  |  | N |
| Echinodermata | Echinoidea |  |  |  | N |
| Echinodermata | Echinoidea | Camarodonta | Echinidae |  | N |
| Echinodermata | Echinoidea | Cidaroida | Cidaridae | *Stereocidaris nascaensis* | N |
| Echinodermata | Echinoidea | Cidaroida | Cidaridae | *Stylocidaris* sp. | N |
| Echinodermata | Holothuroidea |  |  |  | N |
| Echinodermata | Ophiuroidea |  |  |  | N |
| Mollusca | Cephalopoda | Octopoda |  |  | N |
| Porifera |  |  |  |  | Y |
| Porifera | Demospongiae |  |  |  | Y |
| Porifera | Hexactinellida |  |  |  | Y |
|  |  |  |  | Msp1 | N |
|  |  |  |  | Msp2 | N |
|  |  |  |  | Msp3 | N |
|  |  |  |  | Msp4 | N |
|  |  |  |  | Msp5 | N |
|  |  |  |  | Mso6 | N |
|  |  |  |  | Msp7 | N |
|  |  |  |  | Msp8 | N |
|  |  |  |  | Msp9 | N |
