# Supplementary material for: Deep-sea biodiversity at the extremes of the Salas y Gómez and Nazca ridges with implications for conservation
Source: PLoS One. 2021 Jun 30;16(6):e0253213. doi: 10.1371/journal.pone.0253213 (PMC8244922; doi:10.1371/journal.pone.0253213)
Supplement: S3 Table — Msp = morpho-species. * = endemic species. (DOCX) [file pone.0253213.s003.docx]

**S3 Table.** Vertebrate taxa observed on deep-sea camera deployments along the Salas y Gómez and Nazca ridges. Msp = morpho-species.

| Class | Order | Infraorder/Family | Taxa |
| --- | --- | --- | --- |
| Actinopterygii |  |  |  |
| Actinopterygii |  |  | Msp10 |
| Actinopterygii |  |  | Msp11 |
| Actinopterygii |  |  | Msp12 |
| Actinopterygii |  |  | Msp13 |
| Actinopterygii | Anguilliformes |  | Anguilliformes |
| Actinopterygii | Anguilliformes |  | Congridae |
| Actinopterygii | Anguilliformes | Halosauridae | Aldrovandia |
| Actinopterygii | Anguilliformes | Muraenidae | *Gymnothorax bathyphilus* |
| Actinopterygii | Anguilliformes | Nettastomatidae | Nettastomatidae |
| Actinopterygii | Anguilliformes | Nettastomatidae | *Nettastomatidae* sp. |
| Actinopterygii | Anguilliformes | Synaphobranchidae | cf. *Synaphobranchus affinis* |
| Actinopterygii | Anguilliformes | Synaphobranchidae | *Synaphobranchus brevidorsalis* |
| Actinopterygii | Anguilliformes | Synaphobranchidae |  |
| Actinopterygii | Aulopiformes | Ipnopidae | *Bathypterois* |
| Actinopterygii | Aulopiformes | Synodontidae | *Synodus* sp. |
| Actinopterygii | Beryciformes | Berycidae | *Beryx splendens* |
| Actinopterygii | Beryciformes | Monocentridae | *Monocentris reedi* |
| Actinopterygii | Gadiformes |  |  |
| Actinopterygii | Gadiformes | Macrouridae |  |
| Actinopterygii | Gadiformes | Macrouridae | Macrouridae sp. 1 |
| Actinopterygii | Gadiformes | Macrouridae | Macrouridae sp. 2 |
| Actinopterygii | Gadiformes | Moridae | *Antimora rostrata* |
| Actinopterygii | Gadiformes | Moridae | *Laemonema* |
| Actinopterygii | Gadiformes | Moridae |  |
| Actinopterygii | Myctophiformes | Myctophidae |  |
| Actinopterygii | Notacanthiformes | Halosauridae |  |
| Actinopterygii | Ophidiiformes | Ophidiidae | *Spectrunculus* |
| Actinopterygii | Osmeriformes | Alepocephalidae |  |
| Actinopterygii | Perciformes | Carangidae | *Pseudocaranx dentex* |
| Actinopterygii | Perciformes | Carangidae | *Seriola lalandi* |
| Actinopterygii | Perciformes | Carangidae | *Pseudocaranx chilensis* |
| Actinopterygii | Perciformes | Chaetodontidae | *Prognathodes* sp. nov. |
| Actinopterygii | Perciformes | Cheilodactylidae | *Nemadactylus gayi* |
| Actinopterygii | Perciformes | Emmelichthyidae |  |
| Actinopterygii | Perciformes | Epigonidae |  |
| Actinopterygii | Perciformes | Labridae | *Bodianus unimaculatus* |
| Actinopterygii | Perciformes | Labridae |  |

**S3 Table.** Continued. Vertebrate taxa observed on deep-sea camera deployments along the Salas y Gómez and Nazca ridges. Msp = morpho-species.

| Class | Order | Infraorder/Family | Taxa |
| --- | --- | --- | --- |
| Actinopterygii | Perciformes | Lutjanidae | *Etelis carbunculus* |
| Actinopterygii | Perciformes | Lutjanidae | *Parapristipomoides squamimaxillaris* |
| Actinopterygii | Perciformes | Pentacerotidae | *Pentaceros* |
| Actinopterygii | Perciformes | Pinguipedidae |  |
| Actinopterygii | Perciformes | Pomacentridae | *Chromis mamatapara* |
| Actinopterygii | Perciformes | Priacanthidae | *Cookeolus japonicus* |
| Actinopterygii | Perciformes | Serranidae | *Caprodon longimanus* |
| Actinopterygii | Perciformes | Serranidae | *Plectranthias nazcae* |
| Actinopterygii | Perciformes | Serranidae | *Tosanoides* sp. |
| Actinopterygii | Pleuronectiformes | Paralichthyidae |  |
| Actinopterygii | Pleuronectiformes | Paralichthyidae | *Paralichthys fernandezianus* |
| Actinopterygii | Polymixiiformes | Polymixiidae |  |
| Actinopterygii | Scombriformes | Gempylidae | cf. *Rexea* |
| Actinopterygii | Scombriformes | Gempylidae | *Ruvettus pretiosus* |
| Actinopterygii | Scorpaeniformes | Scorpaenidae |  |
| Actinopterygii | Scorpaeniformes | Sebastidae | *Helicolenus* |
| Actinopterygii | Scorpaeniformes | Sebastidae |  |
| Actinopterygii | Scorpaeniformes | Triglidae | *Pterygotrigla picta* |
| Actinopterygii | Stomiiformes | Sternoptychidae | *Argyripnus* |
| Elasmobranchii |  |  |  |
| Elasmobranchii | Hexanchiformes | Hexanchidae | *Hexanchus griseus* |
| Elasmobranchii | Squaliformes | Echinorhinidae | *Echinorhinus cookei* |
| Elasmobranchii | Squaliformes | Etmopteridae |  |
| Elasmobranchii | Squaliformes | Etmopteridae | *Etmopterus* sp. |
| Elasmobranchii | Squaliformes | Squalidae | *Squalus* |
| Elasmobranchii | Squaliformes | Squalidae | *Squalus* cf. *mitsukurii* |
| Holocephali | Chimaeriformes | Chimaeridae | *Hydrolagus* |
| Holocephali | Chimaeriformes | Chimaeridae | *Hydrolagus melanophasma* |
